# Clinical and magnetic resonance imaging monitoring in progressive multifocal leukoencephalopathy treated with pembrolizumab: a case report

**DOI:** 10.1007/s10072-020-04582-4

**Published:** 2020-07-22

**Authors:** Jakob Stögbauer, Walter Schulz-Schaeffer, Ruben Mühl-Benninghaus, Piergiorgio Lochner

**Affiliations:** 1 Department of Neurology, Saarland University, D-66421 Homburg, Saarland Germany; 2 Department of Neuropathology, Saarland University, D-66421 Homburg, Saarland Germany; 3 Department of Neuroradiology, Saarland University, D-66421 Homburg, Saarland Germany

Dear Dr. Federico,

The development of progressive multifocal leukoencephalopathy (PML) under immunosuppressive therapy using CD20 inhibitors such as rituximab is still a rarely observed event in everyday clinical practice and requires increased attention. Furthermore, there is still no causal and evidence-based therapy for the disease. In the recent past, several cases of successful therapy attempts using the programmed cell death 1 protein-inhibitor (PD1) pembrolizumab have been described [1].

In this report, we present the case of a 54-year-old woman who developed an infratentorial PML as a result of combined rituximab, cyclophosphamide, hydroxydaunorubicin, vincristine, and prednisolone (R-CHOP) therapy and in whom three doses of pembrolizumab showed no clinical improvement.

The patient was originally diagnosed with diffuse large cell B cell lymphoma of the non-germinal center B cell (non-GCB)–like type of the small intestine in December 2018. A combined therapy of surgical resection and intravenous chemotherapy was performed using 6 cycles of R-CHOP resulting in complete clinical remission. In November 2019, she was introduced to our clinic with a newly occurring weakness of the right hand, a speech disorder, and dizziness. A neurological examination revealed hemiataxia of the right side of the body. Laboratory tests (including inflammatory indices) were unremarkable. A cerebral magnetic resonance imaging (cMRI) showed a lesion in the area of the right cerebellar hemisphere resembling an edema, without contrast enhancement (Fig. 1, at admission)*.* The secondary involvement of the CNS by DLBCL was excluded because no evidence of clonal B cell population in flow cytometry of the sample of the lumbar puncture was found. Furthermore, cMRI showed neither diffusion restriction nor contrast enhancement which would contribute to the diagnosis of CNS Lymphoma. The microbiological findings were unremarkable. The virological analysis revealed John Cunningham virus (JCV) in the aspirate finally leading to the diagnosis of PML.

**Fig. 1:**
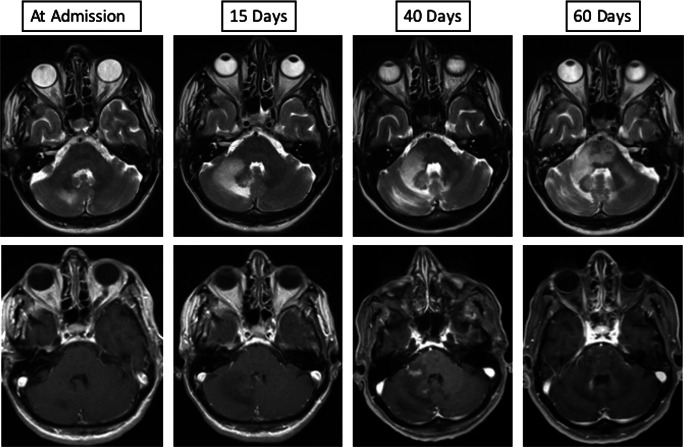
Cerebral magnetic resonance imaging (cMRI) findings. Upper row shows progressive hyperintensity of the infratentorial white matter on axial T2 images representing increase of parenchymal edema (left to right images). Lower row shows axial T1 contrast-enhanced images of the first enhancement of the infratentorial white matter at day 40 after initial imaging that represents the switch from progressive multifocal leukoencephalopathy (PML) to PML immune reconstitution inflammatory syndrome (IRIS) as a radiological finding

Afterwards, we initiated an individual healing attempt with an off-label therapy using mirtazapine with daily target dose of 45 mg (three times/day) and cidofovir infusions. A cMRI revaluation (Fig. 1, 15 days) showed a clear extension of the PML lesion. A second lumbar puncture showed an inflammatory process with a lymphocytic pleocytosis and a high content of cytotoxic T-cells. Consistent with these findings, a florid infection with an increase in the JC viral load by more than two log levels to 3,500,000 copies/ml was determined by quantitative PCR (Fig. 2). In order to restore the immune function, we further escalated the therapy adding the PD1 inhibitor pembrolizumab with the aim of activating JC virus-specific T cells using the same scheme Cortese et al. described (2 mg/kg of body weight, every 4 weeks; altogether three infusions) [1]. Unfortunately, the symptoms of the patient worsened with increasing neurogenic dysphagia, a progressive dysmetria and paresis of the right side in spite of a second infusion of pembrolizumab. A follow-up MRI revealed a further progression of the defect areas including bilateral parts of the brain stem (Fig. 1, 40 days). Despite our efforts, the patient’s condition continued to deteriorate even after the third administration of pembrolizumab. The patient underwent meanwhile a tracheostoma and a percutaneous endoscopic gastrostomy. The last cMRI showed again a progression of the PML lesions with patchy contrast enhancement suggestive of immune reconstitution inflammatory syndrome (IRIS) (Fig. 1, 60 days). Clinically, apart from anarthria, dysphagia, and tetraparesis, the patient had a nearly complete horizontal as well as vertical ophthalmoparesis. Due to the pronounced clinical deterioration, palliative therapy was initiated under which the patient died within 10 days.

**Fig. 2:**
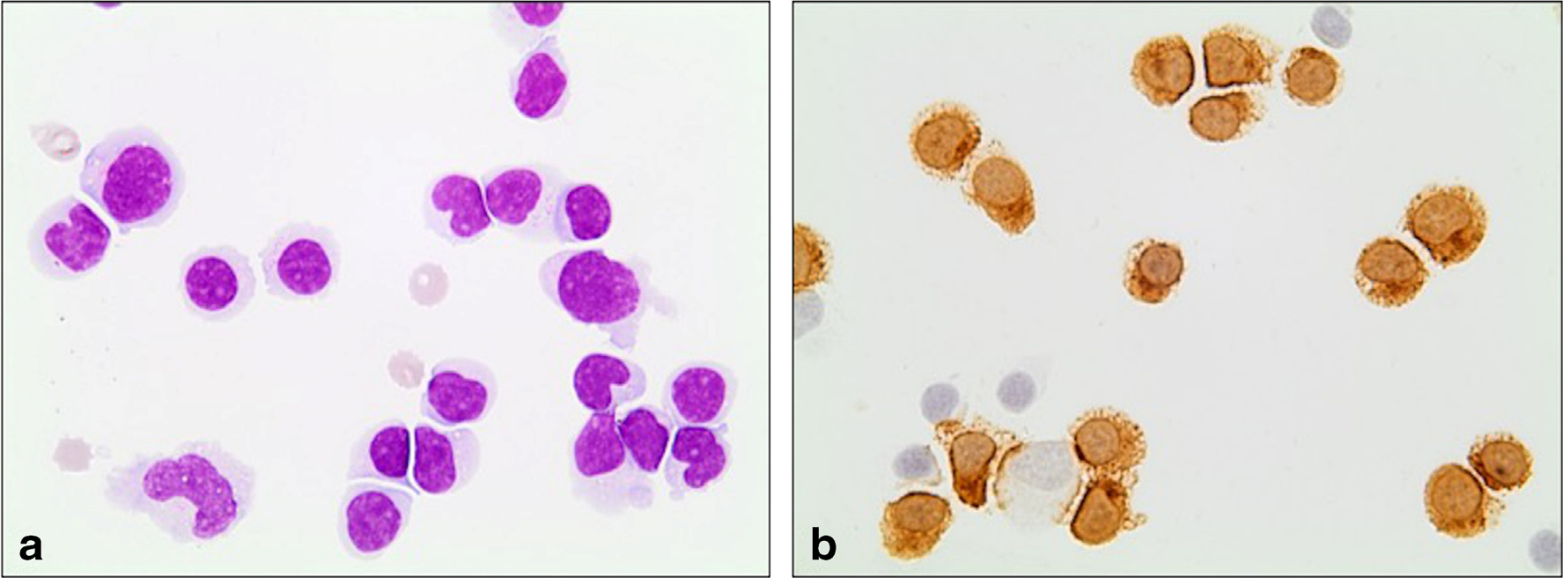
Microscopic images of cerebrospinal fluid cells. **a** Lymphocytic pleocytosis and cell activation, May-Grünwald Giemsa stain. **b** Note the high amount of cytotoxic T-Zells (approx. 70%, CD8-immunoreaction in brown (magnification ×100)

For a long time, PML has been observed almost exclusively as a rare opportunistic infection in the context of Acquired Immune Deficiency Syndrome (AIDS) [2]. Parallel to the increasing clinical use of monoclonal antibodies in the therapy of malignancies and autoimmune diseases such as multiple sclerosis, the incidence of PML has visibly risen. While the risk is well known when patients are treated with the integrin α4 inhibitor natalizumab (Tysabri®) wherefore a routine check of the JC virus status is carried out prior to administration, many people are unaware of the existing potential of a PML manifestation when patients are treated with the CD20 antibody rituximab. The PML is still considered as a very rare side effect of rituximab therapy and occurs in less than 1:10,000 patient cases, although several studies report a significantly higher incidence [3]. Cases similar to this one show that the possibility of PML in patients affected by non-Hodgkin-lymphoma and treated with monoclonal antibodies should be considered in the context of an appropriate clinical syndrome. A prophylactic review of JC serostatus prior to therapy with CD20 inhibitors remains to be discussed. There is still no evidence-based curative therapy for PML and the existing recommendations are usually derived from individual case studies. The most common therapeutic approach is a direct therapy approach using the antiviral drug cidofovir in combination with the tetracyclic antidepressant mirtazapine. Recently the possibility of immunization using a JCV capsid protein (VP1) in combination with interleukin 7 and imiquimod (a toll-like receptor 7 agonist) has also been described in isolated cases [4]. However, so far, this treatment option is not suitable for patients who developed PML as a result of therapy with a monoclonal antibody and are therefore unable to achieve immediate immune reconstitution. In 2019, Cortese et al. discussed an alternative, immunomodulatory treatment option using the PD1 inhibitor pembrolizumab, so far approved for the therapy of several advanced tumor diseases [1]. The new approach pursues the activation of JC virus–specific CD4+ T cells in order to reinvigorate anti-JC virus immune activity. The study included a group of eight patients with PML, five of whom were clinically stabilized or even benefited under pembrolizumab. Subsequently, further therapy trials with the PD1 inhibitor were described, which paint a mixed picture [5, 6]. Some case reports draw a positive picture, in others, as in the present case, no positive effect on the course of the disease could be shown.

At this point in time, no indicator of a patient’s response to therapy could be shown, but a clinical arrest of the disease was observed in some patients treated with PD1 inhibitors. Neither the initial JC viral load in the CSF, nor the time interval between diagnosis and first administration, nor the patient’s age seems to be a reliable prognostic factor [5].

In conclusion, more patients treated with PD1 inhibitors are warranted to prove if and in which stage of the disease PD1 inhibitors should be administered in therapy of patient with PML.

## Data Availability

Not applicable.
